# Global characteristics and drivers of sodium and aluminum concentrations in freshly fallen plant litter

**DOI:** 10.3389/fpls.2023.1174697

**Published:** 2023-06-13

**Authors:** Yiqing Wang, Fuzhong Wu, Qiqian Wu, Kai Yue, Ji Yuan, Chaoxiang Yuan, Yan Peng

**Affiliations:** ^1^ Key Laboratory for Humid Subtropical Eco-Geographical Processes of the Ministry of Education, School of Geographical Sciences, Fujian Normal University, Fuzhou, China; ^2^ State Key Laboratory of Subtropical Silviculture, Zhejiang A&F University, Hangzhou, Zhejiang, China

**Keywords:** mycorrhizal association, leaf form, soil property, climate, meta-analysis, litter quality, life form

## Abstract

Plant litter is not only the major component of terrestrial ecosystem net productivity, the decomposition of which is also an important process for the returns of elements, including sodium (Na) and aluminum (Al), which can be beneficial or toxic for plant growth. However, to date, the global characteristics and driving factors of Na and Al concentrations in freshly fallen litter still remain elusive. Here, we evaluated the concentrations and drivers of litter Na and Al with 491 observations extracted from 116 publications across the globe. Results showed that (1) the average concentrations of Na in leaf, branch, root, stem, bark, and reproductive tissue (flowers and fruits) litter were 0.989, 0.891, 1.820, 0.500, 1.390, and 0.500 g/kg, respectively, and the concentrations of Al in leaf, branch, and root were 0.424, 0.200 and 1.540 g/kg, respectively. (2) mycorrhizal association significantly affected litter Na and Al concentration. The highest concentration of Na was found in litter from trees associated with both arbuscular mycorrhizal fungi (AM) and ectomycorrhizal fungi (ECM), followed by litter from trees with AM and ECM. Lifeform, taxonomic, and leaf form had significant impacts on the concentration of Na and Al in plant litter of different tissues. (3) leaf litter Na concentration was mainly driven by mycorrhizal association, leaf form and soil phosphorus concentration, while leaf litter Al concentration was mainly controlled by mycorrhizal association, leaf form, and precipitation in the wettest month. Overall, our study clearly assessed the global patterns and influencing factors of litter Na and Al concentrations, which may help us to better understand their roles in the associated biogeochemical cycles in forest ecosystem.

## 1 Introduction

Plant litter plays an irreplaceable role in carbon (C) storage and nutrient supply for terrestrial ecosystems, and it is also the main source of soil organic matter and nutrients that affect ecosystem biogeochemical cycles (Vasconcelos and Luizao, 2004). The recycling of nutrients associated with plant litter decomposition is one of the most important ecological processes, and is closely controlled by litter decomposition process (Mooshammer et al., 2012). Sodium (Na) and aluminum (Al) are important nutrients for plant growth (Shen et al., 2020), and the decomposition of litter is an important source of Na and Al. Their concentrations in freshly fallen litter can not only regulate the decomposition process, but also affect ecosystem biogeochemical cycling. However, till now, most of the studies mainly focused on litter decomposition rate, mass loss, and macronutrients such as nitrogen (N) and phosphorus (P), with little research on the initial concentrations of trace elements such as Na and Al (Berg, 2014).

The initial concentrations of plant elements are closely related to soil element status, plant nutrients and resorption efficiency, and can further influence the quality and rate of nutrient return (Tong et al., 2021). Sodium generally exists in plant body in ionic states and plays extremely important roles in regulating osmotic pressure and promoting photosynthesis (Haro et al., 2010). Sodium ion is involved in the formation of chlorophyll in many C_4_ plants and has a stimulating effect on plant growth, but it is easy to be lost because of leaching effects (Laskowski et al., 1995). Low Na concentration may have stimulatory effects on microbial activity simply by acting as a buffer (Frankenberger Jr and Bingham, 1982) to promote the decomposition of plant litter (Kaspari et al., 2014). Aluminum is an ash element of plants, and it has strong impacts on plant physiological activities such as organic acid secretion, cell activity, enzyme activity, and photosynthesis (Panda et al., 2009). Aluminum can combine with components such as glia and proteins in plant cell walls, thereby reducing the elasticity and water conductivity of the cell wall and affecting plant growth (Yoshimura et al., 2003). Most of the Al in nature exists in the form of silicate and Al oxide that are not available for biological use. Therefore, Al from plant litter would be an important source in soils. However, till now, the concentrations of Na and Al in freshly fallen litter across the globe have not been quantitatively assessed, which limits our understanding on their role in litter decomposition and the associated biogeochemical cycling processes.

The concentrations of plant litter Na and Al may be affected by plant functional types (PFT), climate, and soil properties. Element composition differs strongly between plant organs (Minden and Kleyer, 2014). For example, leaf and roots are the main plant tissues for carbon assimilation and nutrient absorption, respectively. Taxonomy and leaf form may also be important driving factors, because they represent similar morphological appearance, structure and habits of plants due to their long-term adaptation to environments, which will affect the element composition in their bodies. The association of mycorrhizal fungi would be also important for accelerating the release of nutrients from litter and improving plant nutrient uptake (Johnson et al., 2016). Mycorrhizal associations may thus indirectly affect trace element concentrations in litters by influencing microbial communities and soil properties (Heděnec et al., 2020). However, how PFT may affect the concentrations of Na and Al in plant litter still remains elusive.

Climatic variables, such as mean annual temperature (MAT) and mean annual precipitation (MAP), may be important drivers of leaf litter Na and Al concentrations (Ge and Xie, 2017), because they are closely related to plant growth and the associated assimilation of elements.(Monroy et al., 2016). Soil properties play an important role in controlling the stoichiometry of plant litters. For example, soil pH and nutrient concentration are comprehensive indicators of soil that can affect plant nutrient uptake efficiency and thus regulate litter element concentration (Tong et al., 2021). Nitrogen concentration is one of the most important factors regulating plant growth, thus may indirectly regulating the absorption, distribution and concentration of plant litter Na and Al (Chen et al., 2021). However, there is still insufficient knowledge on how climate and soil properties may affect the concentrations of Na and Al in freshly fallen plant litter at the global scale.

Here, by compiling a database with 491 observations collected from 116 publications, we assessed the global patterns and driving factors of litter Na and Al concentrations. The objectives of this study were to (1) explore the global patterns of initial Na and Al concentrations in plant litter, including leaf, branch and root litters, and (2) assess the potential impacts of PFT, climate, and soil properties on litter Na and Al concentrations. We hypothesized that (1) the average Na and Al concentrations in leaf, flower and fruit tissues were higher than those in branch, rootbark, and stem, and (2) litter Na and Al concentrations were jointly controlled by mycorrhizal association, leaf form, taxonomic, climate, and soil properties.

## 2 Materials and methods

### 2.1 Literature search and dataset construction

**Figure 1:**
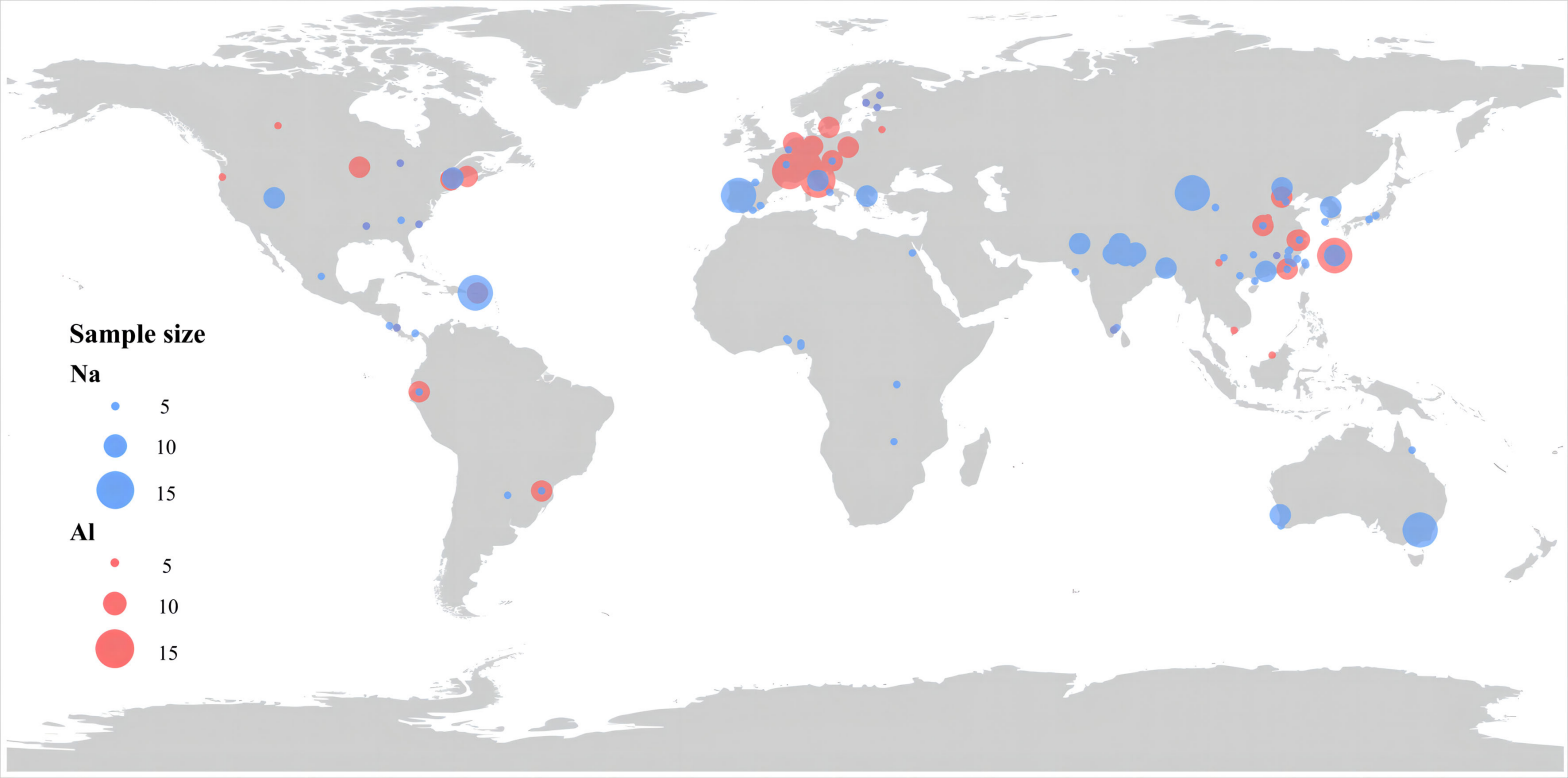
Distribution and sample size of the observations in this study. The sample size for each observation site is represented by symbol size.

We searched Peer-reviewed articles, book chapters, and academic theses that reported Na and Al concentrations of freshly fallen plant litter on October 20, 2021 with China National Knowledge Infrastructure (CNKI) and ISI Web of Knowledge, using the search terms of (sodium OR aluminum OR Na OR Al OR “trace element” OR “metal element”) AND (“plant litter” OR “plant detritus” OR “deadwood” OR “plant residue”). Data were obtained from table, main text, and/or appendices of the primary studies. If the data were presented in figures, the relevant data are obtained by using get data graph digitizer 2.21 software (http://www.getdata-graph-digitizer.com). To be included in our database, we used the following criteria: (1) data for Na or Al concentrations must be measured directly rather than estimated from, for example, statistical models; (2)the Latin names of the plants from which litter were collected must be clearly reported; and (3) litter must be obtained from plants under natural conditions without any treatment such as warming, nitrogen addition, or elevated CO_2_, and plant litter must be collected with nets above forest floors, namely litter from forest floors were not considered here. After extraction, a total of 491data points collected from 116 publications (366 for Na and 125 for Al) were included in our database (**Figure 1**).

**Figure 2:**
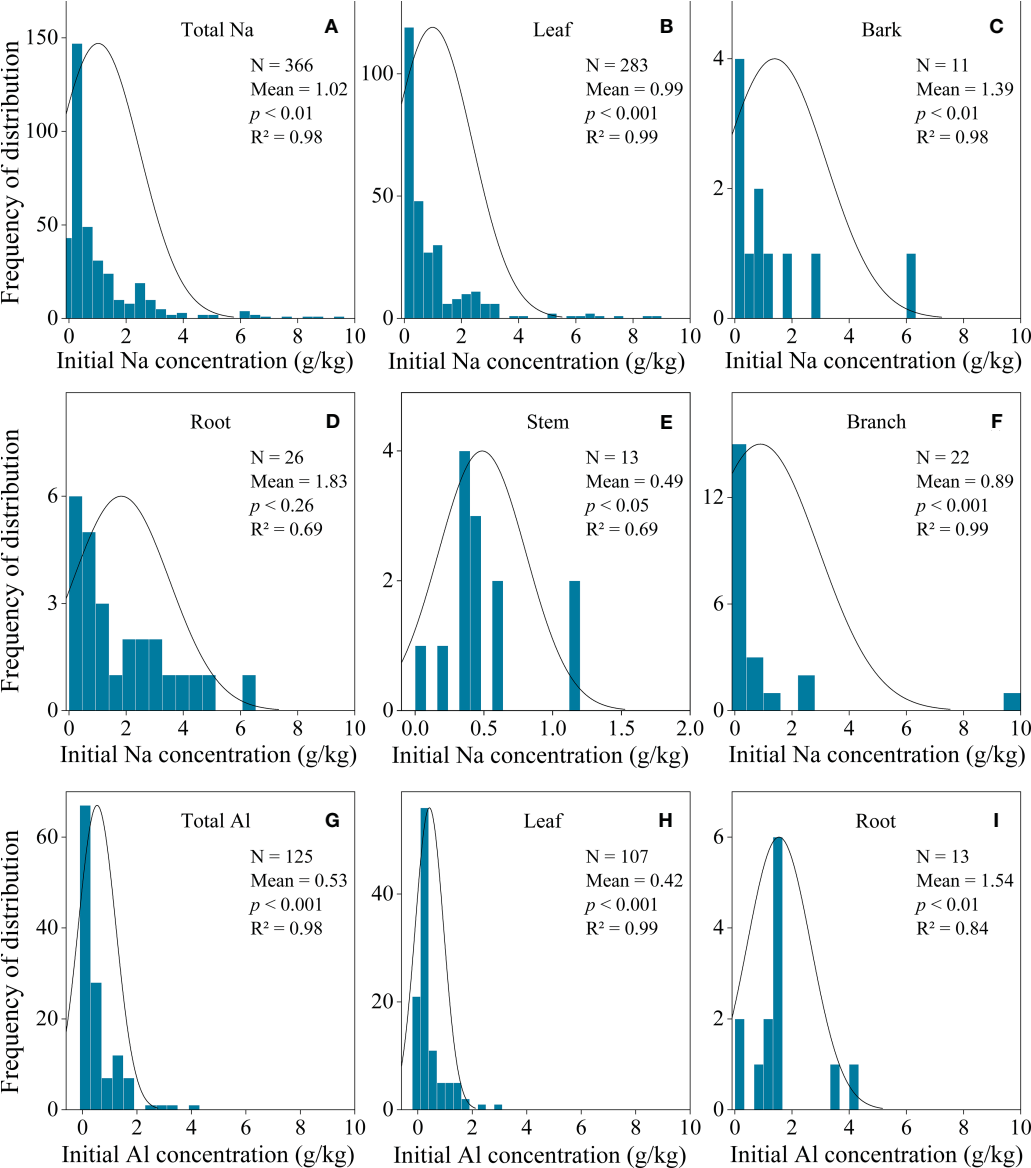
Frequency of the distribution of the initial Na concentrations in total **(A)**, bark litter **(B)**, leaf litter **(C)**, root litter **(D)**, stem litter **(E)**, and branch **(F)**. Frequency of the distribution of the initial Al concentrations in total **(G)**, leaf litter **(H)**, and root litter **(I)**. The numbers and means of each tissue are also shown.

To explore the potential driving factors of litter Na and Al concentrations, we also collected data for geographic location (latitude and slope), mycorrhizal association (AM, ECM and Dual), lifeform (herb, shrub, tree and vine), leaf form (board leaf and conifer) and litter type (leaf, bark, branch, root, stem, flower and fruit) (**Figure 2**), where available. Mycorrhizal association was classified into three types, i.e., arbuscular mycorrhiza (AM), ectomycorrhiza (ECM), and dual (plants associated with both AM and ECM fungi) based on a peer-reviewed database (Soudzilovskaia et al., 2020), and determined lifeform as herbs, shrubs and trees according to previous research (Arianoutsou et al., 2010). Because data for climate and soil properties were not reported in all the primary studies, we thus extracted mean annual temperature (MAT), maximum temperature of the warmest month (TMax), minimum temperature of the coldest month (TMin), mean annual precipitation (MAP), precipitation of the wettest month (WMP), and precipitation of the driest month (DMP) from *WorldClim* v.2.0 (Fick and Hijmans, 2017), and obtained total soil carbon (TOC), soil organic carbon (SOC), total nitrogen (TN), total phosphorus (TP), bulk density, and moisture from SoilGrids 2.0 (Shangguan et al., 2014) based on geographical information of each site.

### 2.2 Statistical analysis

All the statistical analyses were performed in R version 4.0.3. Before statistical analysis, we checked normality and homogeneity of the data, and logarithmically transformed where necessary. We used linear mixed-effects models to evaluate the effects of litter type, climate, soil properties, and geographical locations on litter Na and Al concentrations using the *lme4* package (Bates et al., 2014), and each predictor variable was assessed individually. Because of the limited data points for stem, bark, reproductive tissue, and wood, we only performed these analyses for leaf, branch, and root litter. Then, for the variables that showed significant effects on litter Na and Al concentrations, we used linear mixed-effects model selection method to explore the most important variables using the *glmulti* package based on the maximum likelihood estimation (Calcagno and de Mazancourt, 2010). The importance of each predictor variable was estimated as the sum of Akaike weights of all models containing it (Wagenmakers and Farrell, 2004). The cutoff value of Akaike weight was set to 0.8 following previous research (Yue et al., 2022), which was used to determine the most important predictors of litter Na and Al concentrations.

## 3 Results

### 3.1 Patterns of litter Na and Al concentrations

**Figure 3:**
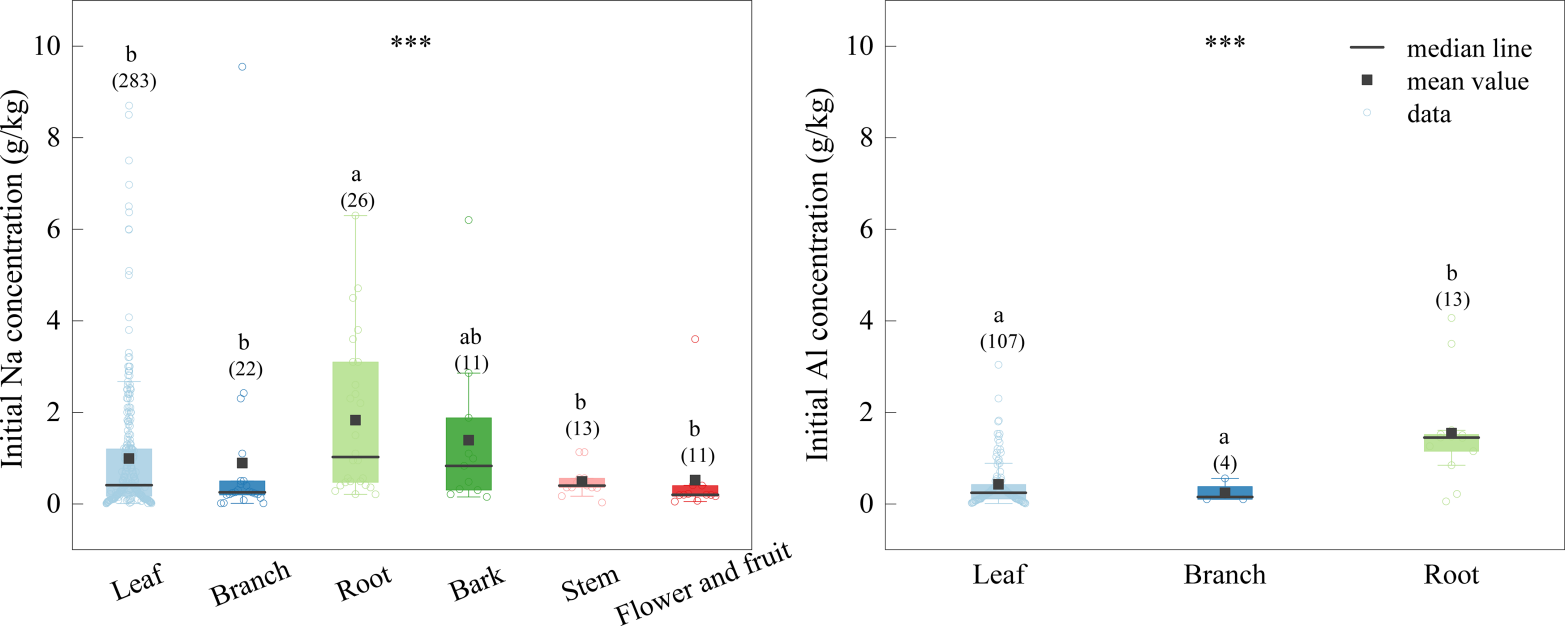
Initial Na and Al concentrations of different tissues. The boxplots show the median and interquartile ranges of the values, the black dots represent the mean value. Different letters indicate significant differences among the different tissues (p < 0.05). The data of initial Al of litter is too little, therefore, we only did comparative analysis of leaf, branches and roots. *** indicate the differences between different tissues, ***p < 0.001.

The concentrations of Na and Al in root litter were significantly higher than those in other litter types, which were 1.820, and 1.543 g/kg, respectively (**Figure 3**). The concentrations of Na in leaves, branches, stems, bark and reproductive tissues were 0.989, 0.891, 0.500, 1.390, and 0.500 g/kg, respectively, and Al concentrations in leaves and branches were 0.424, and 0.200 g/kg, respectively (**Figure 3**).

### 3.2 Driving factors of litter Na and Al concentrations

**Figure 4:**
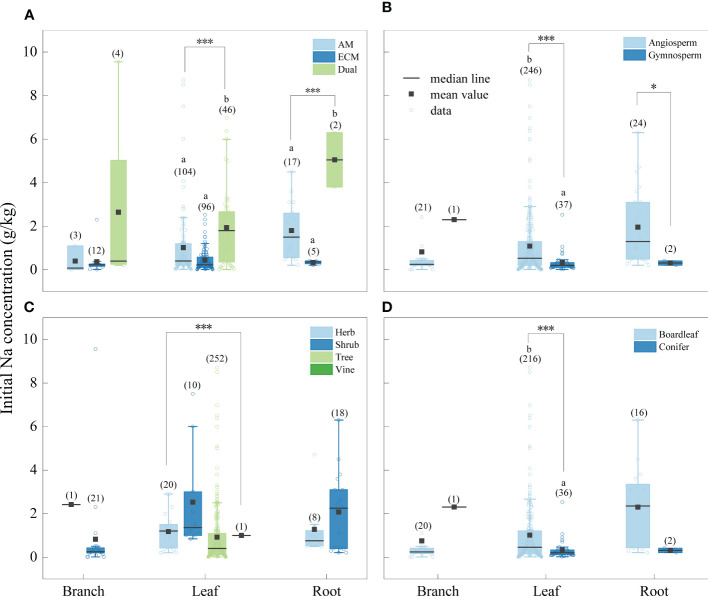
Initial Na concentrations of different tissues (leaf litter, branch litter and root litter) under different mycorrhizal associations **(A)** (AM vs. ECM vs Dual), division **(B)** (angiosperm vs. gymnosperm), life forms **(C)** (herb vs. shrub vs. tree vs. vine) and leaf form **(D)** (board leaf vs. conifer). The boxplots show the median and interquartile ranges of the values, the black dots represent the mean value. Asterisks indicate effects differences at *p < 0.05, ***p < 0.001, and different letters indicate significant differences between each group at the 0.05 level, and the numbers in parentheses represent sample size.

**Figure 5:**
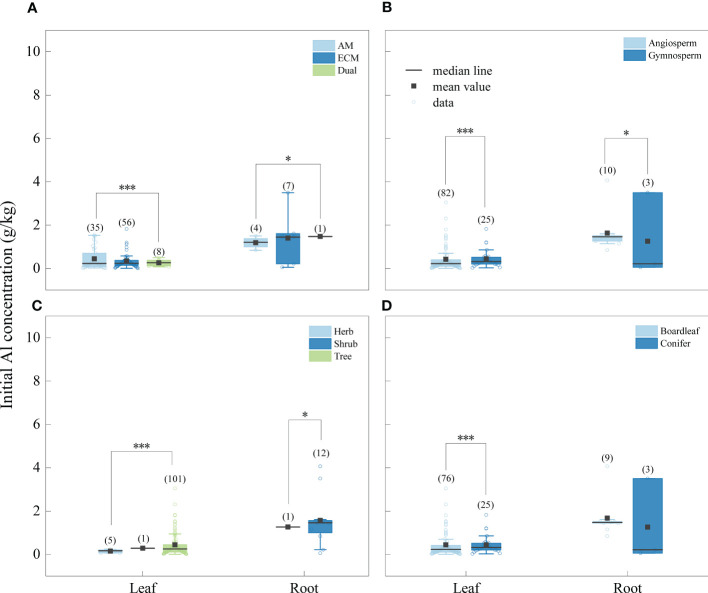
Initial Al concentrations of different tissues (leaf litter and root litter) under different mycorrhizal associations **(A)** (AM vs. ECM vs Dual), division **(B)** (angiosperm vs. gymnosperm), life forms **(C)** (herb vs. shrub vs. tree) and leaf form **(D)** (board leaf vs. conifer). The boxplots show the median and interquartile ranges of the values, the black dots represent the mean value. Asterisks indicate effects differences at *p < 0.05, ***p < 0.001, and different letters indicate significant differences between each group at the 0.05 level, and the numbers in parentheses represent sample size.

**Figure 6:**
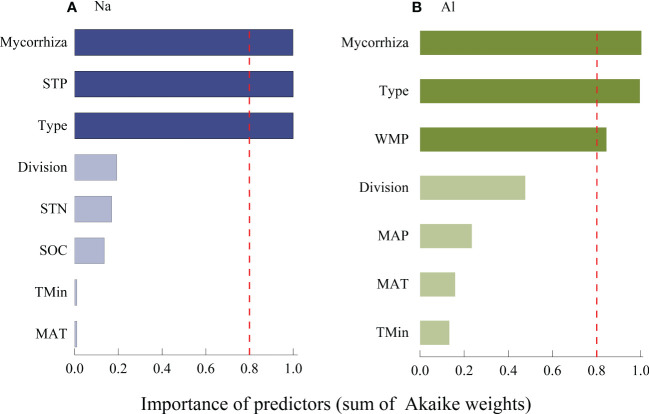
Average importance of plant characteristics, climate and soil properties to the Na **(A)** and Al **(B)** in leaf litter models was assessed using a linear mixed effect model selection method. The virtual line representation cut is set to 0.8 to determine the most important (deep color) predictors. Mycorrhiza, mean types of symbionts between some fungi and plant roots in soil; STP, mean Total soil phosphorus (P); Type, mean leaf form; Division, division of gymnosperms and angiosperms; STN, Total soil nitrogen (N); SOC, Soil organic carbon; TMin, Minimum temperature of the coldest month; MAT, Annual mean temperature; WMP, Precipitation of the wettest month; and AP, Annual precipitation.

The concentration of Na was significantly higher in litter from AM fungi plants than from plants associated with ECM fungi plants (**Figure 4**), and Al concentration was higher in litter from plants associated with AM fungi plants than ECM fungi plants (**Figure 5**). The concentrations of Na and Al were higher in litter from angiosperms than from gymnosperms, from board leaf than from coniferous plants, and from trees than from herbs or shrubs (**Figures 4**, **5**). Leaf litter Na concentration was positively affected by MAT and TMin, but negatively by the concentrations of SOC, STN, and STP (**Table 1**). In contrast, leaf litter Al concentration was only affected by climate, which was positively affected by MAT, TMin, MAP, and WMP. Branch and root litter Na concentration was not affected by climate, soil properties, or geographical location, while branch litter Al concentration was positively affected by MAT and TMax. As to the factors that showed significant impacts, mycorrhizal association, STP, and leaf form were the most important affecting factors for leaf Na concentration, while mycorrhizal association, leaf form, and WMP were the most important for leaf Al concentration (**Figure 6**).

**Table 1:** Linear mixed models were used to evaluate the effects of climate, soil properties and location properties on the initial Na and Al concentration of leaf, branch and root litter.

Predictor	Index	Na			Al		
		Leaf	Branch	Root	Leaf	Branch	Root
Climate	MAT (°C)	0.007**	0.004	0.004	0.003**	0.009*	0.002
	TMax (°C)	0.005	0.002	0.006	0.003	0.007*	0.007
	TMin (°C)	0.004**	0.002	0.002	0.002**	0.424	0.001
	MAP (mm)	0.001	0.001	0.001	0.001*	0.001	0.001
	WMP (mm)	0.001	0.001	0.001	0.001*	0.001	0.001
	DMP (mm)	-0.001	0.001	-0.006	0.001	0.007	0.001
Soil properties	TOC (%)	-0.004	-0.003	-0.001	-0.001	0.016	0.002
	SOC (%)	-0.014**	-0.049	-0.008	-0.001	0.068	0.001
	STN (%)	-0.354**	-0.198	-1.085	-0.039	0.847	0.263
	STP (%)	-0.246***	-0.443	0.091	0.005	0.019	-0.069
	Soil pH	0.014	0.021	0.042	-0.002	-0.085	-0.006
	BD (g/cm^3^)	0.122	0.236	0.212	0.035	-0.706	-0.108
	VWC (%)	-0.002	0.012	0.020*	0.001	0.005	0.004
Location	Slope (°)	-0.002	-0.007	-0.031	0.001	-0.010	-0.003
	Elevation (m)	0.001	0.001	0.000	0.001	0.001	0.001

We regarded each tested factor as a fixed effect and the study identity as a random effect. Estimates and p value are given, and bold indicates statistical significance. Asterisks indicate effects differences at *p < 0.05, **p < 0.01, ***p <0.001.

MAT, mean Annual mean temperature; TMax, mean Maximum temperature of the warmest month; TMin, mean Minimum temperature of the coldest month; MAP, mean Annual precipitation; WMP, mean Precipitation of the wettest month; DMP, mean Precipitation of the driest month; TOC, mean Total soil carbon; SOC, mean Soil organic carbon; STN, mean Total soil nitrogen (N); STP, mean Total soil phosphorus (P); Soil pH, mean soil pH; BD, mean Bulk density; VWC, mean volumetric water concentration at -33 KPa.

## 4 Discussion

We found that the concentrations of Na in root litter were significantly higher than the other litter types. This is potentially due to that Na ions are easier to flow through the cell membrane to promote penetration into the root (Blumwald et al., 2000). And the high discrimination of Na at the soil or root interface often occurs in the stem transport of cations from root to bud, resulting in relatively low Na concentrations in seeds, fruits, and storage tissues of most plant litters (Subbarao et al., 2003). The ability of plant cells to maintain low cytosolic Na concentrations is an essential process associated with the ability of plants to grow in high salt concentrations (Maathuis, 2014). Al ions are mainly absorbed by plants through roots, and only a small amount permeates into leaves (Mossor-Pietraszewska, 2001). The absorption of Al by plant roots is mainly passive, and the absorption is mainly Al ion. Although Al can also move within plants, its mobility is limited, and only a small amount of Al is transferred to the aboveground part, so the Al concentration in the stems and leaves is very low, mainly accumulated in the roots (Alva et al., 1986).

In our study, we discovered that in the initial Na and Al concentrations of leaf litter, Na and Al concentration of AM fungi plants were higher compared to ECM fungi plants. This difference could be attributed to the varying abilities between two mycorrhizal association in the absorption of mineral elements by plants and their transport and accumulation to leaves. Arbuscular mycorrhiza can not only promote the absorption of mineral elements by plants (Allen et al., 1995), but also facilitates transportation to the aboveground parts and enhances the accumulation of mineral elements in plant leaves (Glenn and Gasic, 2015). In contrast, the efficiency of ECM fungi to transfer mineral element is not as good as AM fungi, resulting in lower mineral element concentrations than AM (Shi et al., 2012). In addition, mycorrhizal fungi can lower soil pH by releasing acidic metabolites, thereby promoting the dissolution and release of Na and Al in plant litter (Harley, 1978).

We observed that, except for branch litter, the Na and Al concentration in the litter of other tissues was higher in angiosperms than in gymnosperms. The morphological and structural characteristics of roots in angiosperms and gymnosperms differ significantly (Yahara et al., 2019). The roots of gymnosperms have lower specific root length (SRL) and biological indicator (Bi), indicating that they may be more resource conservative than angiosperms. In contrast, angiosperm roots have higher SRL and Bi, indicating that there are more roots that obtain mineral and nutrients elements (Comas and Eissenstat, 2004). This may be because that the fast-growing fine roots in angiosperms absorb nutrients better than the slow-growing thick roots in gymnosperms (Yang et al., 2022). And angiosperms on the ground typically have wide leaves, while gymnosperms mostly have conifers (Guo et al., 2008), which may also explain why the initial nutrient concentration of board leaf is higher than that of conifers. Furthermore, except for the difference in leaf area between board leaf and coniferous, the reabsorption of nutrients by plants also plays a role in this difference (Deng et al., 2018). Reabsorption enables the litter to complete the internal transfer and reabsorption of nutrients before withering, reducing nutrient loss in the plant. Although board leaf trees also have the characteristics of nutrient reabsorption, the nutrient reabsorption rate of conifers is much higher than that of board leaf trees (Brant and Chen, 2015). Therefore, before the leaves wither and fall to the ground, the conifers with high reabsorption efficiency return most of the nutrients to the plant body, while the board leaf litter retains more nutrients. (Vitousek, 1982).

The transfer of trace elements from nonliving to living compartments of the biosphere is part of the biochemical cycling of chemical elements (Kabata-Pendias, 2004). And plants mainly absorb mineral nutrients from natural soil environment (Haynes, 2012). Consequently, the roots of plants have a large concentration of mineral nutrients, which is consistent with our observation of the extremely high initial Na and Al concentration in the roots of litter. The diffusion of nutrients through the soil to the root system is also affected by soil water. With the increase of precipitation, the effective soil nutrient utilization rate may increase, leading to the increase of Na and Al concentration in plants litter (Cuevas and Medina, 1986). Our results showed that there was a significant negative correlation between soil phosphorus (P) and soil nitrogen (N)on the initial Na concentration of litter. Soil P can affect the dissolution and adsorption process of initial Na concentration in litter, with the higher P concentration, the more Na ions adsorbed and fixed in litter (Eghball et al., 1996). The higher soi N concentration in the soil may increase the number and activity of soil microorganisms, and promoting the decomposition and mineralization of plant litter, releasing elements such as Na, and also reducing the initial Na concentration of plant litter (Gao et al., 2012). But excessive amount of Na concentration can cause soil moisture imbalance, inhibit soil microbial activity, and negatively impact the absorption of nutrients by plants and the decomposition of plant litter (van Huysen et al., 2016). Soil salt stress affects various physiological and metabolic processes and may ultimately influence the concentration of Na in plants litter (Keisham et al., 2018). The high concentration of solute in the soil will lead to osmotic stress, reduce the ability of roots to absorb water, and accelerate the loss of water in leaves. This is accompanied by ion-specific effects, resulting in the accumulation of concentrations of Na ion in plant cells.

Existing studies indicate that climate variables, such as annual average temperature (MAT) and annual average precipitation (MAP), play a significant role in determining litter stoichiometry on a large geographical scale (Erickson et al., 2014). Among these variables, precipitation has the most obvious effect on the nutrient concentration of leaf litter (Cuevas and Medina, 1986). Our observation indicates that precipitation generally affects the initial nutrient concentration of litter through two aspects. Firstly, low precipitation can cause the surface soil to dry up and harden, decreasing the efficiency of litter decomposition and nutrient absorption, and hindering the transfer of soil nutrients to plant litter, resulting in a lower nutrient concentration in leaves (Holbrook et al., 1995). Secondly is that when the precipitation is scarce, the development of plants may be limited, making plants premature aging. Premature senescence may lead to premature withering of plants due to incomplete nutrient absorption, resulting in lower nutrient concentration in leaf litter (Murphy and Lugo, 1986). Therefore, the nutrient concentration of plant litter may be higher in the period of high precipitation than in the period of drought.

## 5 Conclusions

Sodium and Al are crucial elements known to significantly influence plant growth. Understanding the distribution of these elements within different plant tissues and their response to environmental changes is of paramount importance. Our results revealed that the concentrations of Na and Al in root litter was found to be significantly higher compared to other plant tissues. The concentration of Na in leaf litter was primarily driven by factors such as mycorrhizal association, leaf morphology, and soil phosphorus concentration. In contrast, the concentration of Al in leaf litter was predominantly influenced by mycorrhizal association, leaf morphology, and precipitation levels during the wettest month. These findings contribute to our understanding of the intricate mechanisms underlying the distribution of Na and Al within plant tissues. By elucidating the factors governing their concentrations, particularly in root and leaf litter, this research sheds light on the ecological implications of Na and Al dynamics and their potential impacts on plant growth in response to varying climatic and soil conditions.

## Data availability statement

Raw data used in the study were deposited in figshare with a DOI (https://doi.org/10.6084/m9.figshare.22828148.v1).

## Author contributions

YP and YW conceived the study. YW, FW, QW, KY, JY, and CY collected raw data. YW, FW, QW, KY, and YP performed the statistical analyses and wrote the first draft of the manuscript, with contributions from all coauthors. All authors contributed to the article and approved the submitted version.
