# Supplementary material for: Global characteristics and drivers of sodium and aluminum concentrations in freshly fallen plant litter
Source: Front Plant Sci. 2023 Jun 13;14:1174697. doi: 10.3389/fpls.2023.1174697 (PMC10293839; doi:10.3389/fpls.2023.1174697)
Supplement: Supplementary file 1 [file DataSheet_1.pdf]

Supplementary Material

Global characteristics and drivers of sodium and aluminum concentrations in freshly fallen plant litter

Yiqing Wang[1], Fuzhong Wu[1], Qiqian Wu[2], Kai Yue[1], Ji Yuan[1], Chaoxiang Yuan[1], and Yan Peng[1*]

[*]**Corresponding author:** Yan Peng

Email: ypeng117@163.com; yanpeng@fjnu.edu.cn

1 Appendix:

A list of the primary studies from which data were extracted.

1. A, D, Boble., Lrene, Zenneck., P, J, Randall. (1955). Leaf litter ash alkalinity and neutralisation of soil acidity. *Plant and Soil*. 1996(173), 293-302.
2. A, Fioretto., S, Papa., G, Sorrentino., A, Fuggi. (2001). Decomposition of Cistus incanus leaf litter in a Mediterranean maquis ecosystem: mass loss, microbial enzyme activities and nutrient changes. *Soil Biology & Biochemistry*. 33(2001), 311-321.
3. A, Gonzalez-Arias., I. Amezaga., A. Echeandia., M. Domingo., & M, Onaindia. (1998) Effects of pollution on the nutrient return via litterfall for Pinus radiata plantations in the Basque Country. *Plant Ecology*. 189 247-258.
4. A, M, Ocinnell. (1987). Decomposition of Leaf Litter in Karri (Eucalyptus diversicolor) Forest of Varying Age. *Forest Ecology and Management*. 1988(24), 113-125.
5. A, M, Oconnell. (1987). Nutrient Accumulation in and Release from the Litter Layer of Karri (Eucalyptus diversicolor) Forests of Southwestern Australia. *Forest Ecology and Management*. 26(1989), 95-111.
6. A, M, Oconnell. (1988). Nutrient dynamics in decomposing litter in karri (eucalyptus diversicolor f. muell.) forests of south-western Australia. *Journal of Ecolog*. 1988(76), 1186-1203.
7. A, M, Oconnell., P, M, A, menage. (1982). Litter fall and nutrient cycling in karri {Eucalyptus diversicolor F. Muell.) forest in relation to stand age. *Australian Journal of Ecology*. 1982(7), 49-62.
8. A, M, Oconnell., P, Menage. (1983), Decomposition of litter from three major plant

species of jar rah {Eucalyptus marginata Donn ex Sm.) forest in relation to site fire history and soil type. *Australian Journal of Ecology.* 1983(8), 277-286.
9. A, M, Oconnell., T, S, Grove., G, M, Dimmock. (1978). Nutrients in the litter on jarrah forest soils. *Australian Journal of Ecology*. 1978(3), 253-260.
10. A, Smit., A, M, Kooijman. (2000). Impact of grazing on the input of organic matter and nutrients to the soil in a grass-encroached Scots pine forest. F*orest Ecology and Management*. 142(2001) 99-107.
11. Agbim NN (1987) Dry Season Decomposition of Leaf Litter from Five Common Plant Species of West Africa. *Biological Agriculture & Horticulture* 4: 213-224.
12. Akinyele AO, Donald-Amaeshi U (2021) Leaf litter decomposition and nutrient release of three selected agroforestry tree species. *Agroforestry Systems* 95: 559-570.
13. Aklilu, Negussie., Jeroen, Degerickx., Lindesy, Norgrove., Wouter, M, J., Achten., Kiros, Meles, Hadgu., Ermias, Aynekulu., Bart, Muys. Effects of accession, spacing and pruning management on in-situ leaf litter decomposition of Jatropha curcas L. in Zambia.
14. Alexander Heim., Beat, Frey. (2003). Early stage litter decomposition rates for Swiss forests. *Biogeochemistr*. 2004(70), 299-313.
15. Almagro M, Martínez-Mena M (2012) Exploring short-term leaf-litter decomposition dynamics in a Mediterranean ecosystem: dependence on litter type and site conditions. *Plant and Soil* 358: 323-335.
16. Anthony, I, Okeke., C, P, E, Omaliko. (1993). Litterfall and seasonal patterns of nutrient accumulation in Dactyladenia barteria (Hook f ex. Oliv.) Engl. bush fallow at Ozala, Nigeria. *Forest Ecology and Management*. 67(1994), 345-351.
17. Aucina A, Rudawska M, Leski T, Skridaila A, Riepsas E, Iwanski M (2007) Growth and mycorrhizal community structure of Pinus sylvestris seedlings following the addition of forest litter. *Appl Environ Microbiol* 73: 4867-4873.
18. Bas Van WEsemael. (1992). Litter decomposition and nutrient distribution in humus profiles in some mediterranean forests in southern Tuscany. *Forest Ecology and Management*. 1993(57), 99-114.
19. Berger TW, Berger P (2014) Does mixing of beech (Fagus sylvatica) and spruce (Picea abies) litter hasten decomposition? *Plant Soil* 377: 217-234.
20. Chen, W, W., Kou, L., Jiang, L., Gao, W, L., Yang, H., Wang, H, M. Li, S, G. (2017). Short-term responses of foliar multi-element stoichiometry and nutrient resorption of slash pine to N addition in subtropical China. *Chinese Journal of Applied Ecology*. 28(4), 1096-1102. (in Chinese with English abstract).
21. Chen, Y, R. (2000). Nutrient Element Dynamic Analysis of Withered Leaves from Artificial Forest of Masson Pine on the Experimental Plot in Qianyanzhou. JIANGXI SCIENCE. 18(1), 19-23, (in Chinese with English abstract).
22. Chen, Y. (2003). Study on Litter-fall Decomposing Dynamic and Nutrients Releasing rule of phoebe bournet. (in Chinese with English abstract).
23. Cizungu L, Staelens J, Huygens D, Walangululu J, Muhindo D, Van Cleemput O, Boeckx P (2014) Litterfall and leaf litter decomposition in a central African tropical mountain forest and Eucalyptus plantation. *Forest Ecology and Management* 326: 109-116.

24. Dale, L, Bartos., Norbert, V, Debyle. (1981) Quantity, Dynamics Decomposition, and Nutrient of Aspen Litterfall in Utah. *Forest science*. 27(2), 381-390.
25. Dr, Victor, A, Kavvadias., Dr, Dimitrios, Alifragis., DR, Alexandoros, Tsiontsis., Dr, Georgios, Brofas., Dr, Georgios, Stamatelos. (2000). Litterfall, litter accumulation and litter decomposition rates in four forest ecosystems in northern Greece. *Forest Ecology and Management*. 144(2001), 113-127.
26. Du B, Ji H, Peng C, Liu X, Liu C (2016) Altitudinal patterns of leaf stoichiometry and nutrient resorption in Quercus variabilis in the Baotianman Mountains, China. *Plant and Soil* 413: 193-202.
27. Duong, Van Ni., To, Phuc, Tuong., Edward, Maltby. (2016). Decomposition of litter from Melaleuca cajuputi affects surface water quality in acid sulphate soils in the Mekong Delta, Vietnam. *Researchgate*. 2016(9).
28. Elon, S, Verry., (1976). Elements in leaves of a trembling aspen clone by crown position and season. *Can.j.for.res*. 1976(6), 436-440.
29. Fan, W., Wang, G, Q. (1992). Study on material cycle of agroforestry complex ecosystem. *Rural ecological environment*. 1992(2), 37-39.
30. Farahat E, Linderholm HW (2015) Nutrient resorption efficiency and proficiency in economic wood trees irrigated by treated wastewater in desert planted forests. *Agricultural Water Management* 155: 67-75.
31. Filippo Bussotti, Francesca, Borghini., Carlo Celesti., Claudio, Leonzio., Alberto, Cozzi., Davide, Bettini., Marco, Ferretti. (2003). Leaf shedding, crown condition and element return in two mixed holm oak forests in Tuscany, central Italy. *Forest Ecology and Management*. 176(2003) 173-285
32. Fioretto A, Papa S, Fuggi A (2003) Litter-fall and litter decomposition in a low Mediterranean shrubland. *Biology and Fertility of Soils* 39: 37-44.
33. Goloran JB, Chen C, Phillips IR, Elser JJ (2015) Shifts in leaf N:P stoichiometry during rehabilitation in highly alkaline bauxite processing residue sand. *Sci Rep* 5: 14811.
34. Guy, N, Cameron., Stephen, R, Spencer. (1989). Rapid leaf decay and nutrient release in a chinese tallow forest. *Oecologia*. 1989(80), 222-228.
35. H, M, Brasell., D, F, Sinclair. (1983). Elements returned to forest floor in two rainforest and three plantation plots in tropical Australia. *Journal of Ecology*. 1983(71), 367-378.
36. H, Staaf. (1987). Foliage litter turnover and earthworm populations in three beech forests of contrasting soil and vegetation types. *Oecologia (Berlin)*. 1987(72), 58-64.
37. Hu, S, H., Chen, L, Z., Chen, Q, L., Kong, F, S., Liao, Y, G. (1987). Experimental study on decomposition rate of dead leaves of several trees. *Acta phytoecologica et geobotanica sinica*. 11(2),124-132 (In Chinese)
38. Hu, S, H., Chen, L, Z., Kong, F, Z., & Bao, X, Z. (1986) Decomposition rate of dead leaves of two Chinese endemic species. *Acta phytoecologica et geobotanica sinica*. 10(1) 35-43 (In Chinese).
39. Hu, S, H., Chen, L, Z., Kong, F, Z., Ren, J, K. (1986). Study on decomposition of dead leaves of Pinus tabulaeformis and Quercus variabilis. *Acta Botanica Sinica*. 28(1), 102-110.

40. Huang, Y, R., Ye, G, F., Gao, W., Yue, J, X., Nie, S. (2021). Metal Element Content and Reabsorption Rate in Leaves of Main Tree Species in Subtropical Coastal Sandy Land. *Journal of Subtropical Resources and Environment*. 16(2), 10-16.
41. Huangfu C-h, Wei Z-s (2018) Nitrogen addition drives convergence of leaf litter decomposition rates between Flaveria bidentis and native plant. *Plant Ecology* 219: 1355-1368.
42. J, C, Tappeiner., A, A, Alm. (1975). Undergrowth vegetation effects on the nutrient content of litterfall and soils in red pine and birch stands in northern Minnesota. *Ecology*. 1975(56), 1193-1200.
43. Jacques Ranger., Frederic, Gerard., Monika Lindemann., Dominque, Gelhaye., Louisette Gelhaye. (2002), Dynamics of litterfall in a chronosequence of Douglas-fir (Pseudotsuga menziesii Franco) stands in the Beaujolais mounts (France). 60(2003) 457-488.
44. James, A, Entry., Cathy, L, Rose.,Kermit, Cromack, Jr. (1990). Litter decomposition and nutrient release in ectomycorrhizal mat soils of a douglas fir ecosystem. *Soil Bid. Bmckm*. 23(3), 285-290.
45. James, R, Gosz. (1973). Nutrient release from decomposing leaf and branch litter in the hubbard brook forest, new Hampshire. *Ecological Monograph*. 1973(43), 173-191.
46. Jha P, Mohapatra KP, Dubey SK (2014) Fine Roots Carbon Mineralization and Soil Carbon Stabilization Under Major Tree Species of the Semi-arid Region of India. *National Academy Science Letters* 37: 413-418.
47. Ji H, Wen J, Du B, Sun N, Berg B, Liu C (2018) Comparison of the nutrient resorption stoichiometry of Quercus variabilis Blume growing in two sites contrasting in soil phosphorus content. *Annals of Forest Science* 75.
48. Joseph, Ikechukwu, Muoghalu., Olalekan, Mujahid, Adeleye., Rotimi, Tunde, Balogun. (1994). Litter decomposition and inorganic element dynamics in a secondary rainforest at Ile-Ife, *Nigeria. Afr. J. Ecol*. 1994(32), 208-221.
49. Juan, F, Gallardo., Alejandro, Martin., Lgnacio, Santa, Regina. (1988). Nutrient cycling in deciduous forest ecosystems of the Sierra de Gata mountains: aboveground litter production and potential nutrient return. *Ann Sci For*. 1988(55), 749-769.
50. Lai, C, B., Annapurna, C., Raghubanshi, A, S., Singh, J, S. (2001). Foliar demand and resource economy of nutrients in dry tropical forest species. *Journnl of Vegetation Science*. 2001(12), 5-14.
51. Lee KE, Cha S, Lee SH, Shim JK (2015) Decomposition of leaf litter of some evergreen broadleaf trees in Korea. *Journal of Ecology and Environment* 38: 517-528. doi: 10.5141/ecoenv.2015.054.
52. Lee YC, Nam JM, Kim JG (2010) The influence of black locust (Robinia pseudoacacia) flower and leaf fall on soil phosphate. *Plant and Soil* 341: 269-277.
53. Lehto T, Smolander A, Aphalo PJ (2009) Decomposition and element concentrations of silver birch leaf litter as affected by boron status of litter and soil. *Plant and Soil* 329: 195-208.
54. Li H, Wei Z, Huangfu C, Chen X, Yang D (2017) Litter mixture dominated by leaf litter of the invasive species, Flaveria bidentis, accelerates decomposition and favors

nitrogen release. *J Plant Res* 130: 167-180.
55. Li Y, Xu M, Zou X (2006) Effects of nutrient additions on ecosystem carbon cycle in a Puerto Rican tropical wet forest. *Global Change Biology* 12: 284-293.
56. Li Y, Xu M, Zou X, Shi P, Zhang Y (2005) Comparing soil organic carbon dynamics in plantation and secondary forest in wet tropics in Puerto Rico. *Global Change Biology* 11: 239-248.
57. Li, Z, A., Wang, B, S., Wen, H., Tu, M, Z., & Yao, W, H. (1998) Nutrient dynamics of litterfall in lower subtropical monsoon evergreen broad-leaved forest of dinghushan. *Journal of Tropical and Subtropical Botany*. 6(3): 209-215 (in Chinese with English abstract).
58. Li, Z, W. (2014). Influence of gap on litter decomposition and nutrient dynamics in typical mixed broadleaved-Korean pine forest in Xiaoxing an Mountains. (in Chinese with English abstract).
59. Lim S-M, Cha S-S, Shim J-K (2011) Effects of simulated acid rain on microbial activities and litter decomposition. Journal of Ecology and Environment 34: 401-410.
60. Lin, Y, M., He, J, Y., Yang, Z, W., Liu, C, T., Lin, P., Li, Z, J. (1999). Litter yield and its dynamics of Castanopsis eyrei community in Mount Wuyi. *Journal of Xiamen University*. 38(2), 280-286. (In Chinese)
61. Lin, Y., Zhang, Y, Huang, X, Y., Zhang, S., He, Z, M., Lin, S, Z., Guan, G, D. (2014). Litter Fall and Decomposition of Eucalyptus urophylla Egrandis Plantation in Coastal Sandy Soil. *Journal of northeast forestry university*. 42(3), 11-14.
62. Lindesy, E, Rostad. (1994), Element dynamics along a decay continuum in a red spruce ecosystem in maine, usa. *Ecology*. 75(4), 867-879.
63. Lindsey, E, Rustad., Lvan, J, Fernandez. (1988). Soil Warming: Consequences for Foliar Litter Decay in a Spruce-Fir Forest in Maine, USA. 1998(62), 1072-1080.
64. Liu J, Liu S, Li Y, Liu S, Yin G, Huang J, Xu Y, Zhou G (2017) Warming effects on the decomposition of two litter species in model subtropical forests. *Plant and Soil* 420: 277-287.
65. Liu P, Huang J, Han X, Sun OJ (2009) Litter Decomposition in Semiarid Grassland of Inner Mongolia, China. *Rangeland Ecology & Management* 62: 305-313.
66. Lovett GM, Arthur MA, Crowley KF (2015) Effects of Calcium on the Rate and Extent of Litter Decomposition in a Northern Hardwood Forest. *Ecosystems* 19: 87-97.
67. Luo D, Cheng R, Shi Z, Wang W (2017) Decomposition of Leaves and Fine Roots in Three Subtropical Plantations in China Affected by Litter Substrate Quality and Soil Microbial Community. *Forests* 8.
68. Lv, J, H. (2018). Effects of nitrogen deposition on stoichiometry, nutrient resorption of leaf and nutrient return of leaf litter in a Moso Bamboo forest. (in Chinese with English abstract).
69. M, Liangovan., Kailash, Paliwal. (1994). Changes in mass and nutrients during decomposition of leucaena leucocephala and cymbopogon citratus and the effect of substrate quality, weather variables and soil variables on mass loss during decomposition in a semi-arid ecosystem, madurai, india. *Journal of Tropical Forest Science*. 8(3), 317-332.
70. M, Uma., Ts, Saravanan., K, Rajendran. (2012). Growth, litterfall and litter

decomposition of casuarina equisetifolia in a semiarid zone. *Journal of Tropical Forest Science*. 26(1), 125-133.
71. Marc, Goebel., Sarah, E, Hobble., Bartosz, Bulaj., Marcin, Zadworny., Douglas, D, Archibald., Jacky, Oleksyn., Peter, B, Reich., Davin, M, Ewssenstat. (2011). Decomposition of the finest root branching orders:linking belowground dynamics to fine-root function and structure. *Ecological Society of America*. 81(1), 89-102.
72. Marlin, R, Gottschalk., Donald, J, Shure. (1979). Herbicide effects on leaf litter decomposition processes in an oak-hickory forest. *Ecological Society of America*. 60(1), 143-151.
73. Morrison IK (2003) Decomposition and element release from confined jack pine needle litter on and in the feathermoss layer. *Canadian Journal of Forest Research* 33: 16-22.
74. N, A, Mcenroe., H, S, Helmisaari. (2000). Decomposition of coniferous forest litter along a heavy metal pollution gradient, south-west Finland. *Environmental Pollution*. 2001(113), 11-18.
75. Neiff JJ, de Neiff AP (1990) Litterfall, leaf decomposition and litter colonization of Tessaria integrifolia (compositae) in the Parana river floodplain. *Hydrobiologia* 203: 45-52.
76. Nikula S, Vapaavuori E, Manninen S (2010) Urbanization-related changes in European aspen (Populus tremula L.): leaf traits and litter decomposition. *Environ Pollut* 158: 2132-2142.
77. Nirmal Kumar JI, Kumar RN, Bhoi RK, Patel K (2010) Seasonal changes of bioelements in litter and their potential return to green leaves in five species of tropical dry deciduous forest, western India. *Journal of Forestry Research* 21: 33-38.
78. Ohashi M, Makita N, Katayama A, Kume T, Matsumoto K, Kumagai To, Endo I, Kho LK (2019) Characteristics of root decomposition based on in situ experiments in a tropical rainforest in Sarawak, Malaysia: impacts of root diameter and soil biota. *Plant and Soil* 436: 439-448.
79. Paul, Oconnor., Alan, P, Covich., F, N, Scatena., Lloyd, L, Loope. (2016). Non-indigenous bamboo along headwater streams of the Luquillo Mountains, Puerto Rico: leaf fall, aquatic leaf decay and patterns of invasion. Journal of Tropical *Ecoloy.* 2000(16), 499-516.
80. Pérez-Suárez M, Arredondo-Moreno JT, Huber-Sannwald E (2011) Early stage of single and mixed leaf-litter decomposition in semiarid forest pine-oak: the role of rainfall and microsite. *Biogeochemistry* 108: 245-258. doi: 10.1007/s10533-011-9594-y.
81. Powers JS, Salute S (2011) Macro- and micronutrient effects on decomposition of leaf litter from two tropical tree species: inferences from a short-term laboratory incubation. *Plant and Soil* 346: 245-257.
82. Rawat YS, Singh JS (1988) Structure and Function of Oak Forests in Central Himalaya. I. Dry Matter Dynamics. *Annals of Botany* 62: 397-411.
83. Rebecca, Kershnar., Florencia, Montagnini. (1988). Leaf Litter Decomposition, Litterfall, and Effects of Leaf Mulches from Mixed and Monospecific Plantations in Costa Rica. *Journal of Sustainable Forestry*. 1988, 7(3/4).

84. S, V, Briggs., M, T, Maher. (1983). Litter Fall and Leaf Decomposition in a River Red Gum (Eucalyptus camaldulensis) Swamp. 31, 307-16.
85. Sarker TC, Maisto G, De Marco A, Esposito F, Panico SC, Alam MF, Mazzoleni S, Bonanomi G (2018) Explaining trajectories of chemical changes during decomposition of tropical litter by 13C-CPMAS NMR, proximate and nutrients analysis. *Plant and Soil* 436: 13-28.
86. Sayer EJ, Tanner EVJ, Lacey AL (2006) Effects of litter manipulation on early-stage decomposition and meso-arthropod abundance in a tropical moist forest. *Forest Ecology and Management* 229: 285-293.
87. Sen, Her, Shieh., Chorng, Bin, Hsu., Chiao, Ping, Wang., Ping, Shih, Yang. (2007). Leaf Breakdown in a Subtropical Stream Riffle and Its Association with Macroinvertebrates. *Zoological Studies*. 46(5), 609-621.
88. Singh B (1998) Contribution of forest fine roots in reclamation of semiarid sodic soils. *Arid Soil Research and Rehabilitation* 12: 207-222.
89. Tian, X, J., Sun, Shu, Cun., Ma, Ke, Ping., An, Shu, Q. (2003). Behavior of Carbon and Nutrients Within Two Types of Leaf Litter During 3.5-year Decomposition. *Acta Botanica Sinica*. 45(12), 1413-1420.
90. Trum F, Titeux H, Ponette Q, Berg B (2015) Influence of manganese on decomposition of common beech (Fagus sylvatica L.) leaf litter during field incubation. *Biogeochemistry* 125: 349-358.
91. Trum F, Titeux H, Ranger J, Delvaux B (2011) Influence of tree species on carbon and nitrogen transformation patterns in forest floor profiles. *Annals of Forest Science* 68: 837-847.
92. Tsutomu, Enoki., Hideyuki, Kawaguchi. (2000). Initial nitrogen content and topographic moisture effects on the decomposition of pine needles. *Ecological Research*. 2000(15), 425-434.
93. V P Upadhyay. (1993) Effect of initial litter quality on decomposition rate of tree leaf litter in Himalayan forest ecosystems. 34(1): 44-50.
94. V, P, Upadhyay., J, S, Singh., Meentemeyer. (1989). Dynamics and weight loss of leaf litter in central himalayan forests: abiotic ;i versus litter quality influences. *Journal of Ecology*. 1989(77), 147-161.
95. Vorgelegr, Von., M, Sc., Laura, Margarita, Sanchez, Galindo. (2020). Impacts of leaf litter diversity and root resources on microorganisms and microarthropods (Acari, Collembola) during early stages of decomposition in tropical montane rainforest ecosystems.
96. Wang Q, Kwak J-H, Choi W-J, Chang SX (2018) Decomposition of trembling aspen leaf litter under long-term nitrogen and sulfur deposition: Effects of litter chemistry and forest floor microbial properties. *Forest Ecology and Management* 412: 53-61.
97. Wang, D, F., Herbert, Bormann., Ariel E, Lugo., & Richard, Bowden. (1990) Comparison of nutrient-use efficiency and biomass production in five tropical tree taxa. *Forest Ecologv and Management*. 46(1991) 1-21.
98. Wang, H, L., Zhuang, S, Y., Qian, Z, Z., Cao, J, J. (2019). Leaf litter decomposition dynamics of Pinus taiwanensis and Quercus variabilis associated with various elevations. *Journal of Research in Ecology.* 7(1), 2486-2496.

99. Wang, J., Huang, J, H. (2001). Comparison of major nutrient release patterns in leaf litter decomposition in warm temperate zone of china. Acta Phytoecologica Sinica. 25(3), 375-380. (in Chinese with English abstract).
100. Waring BG (2013) Exploring relationships between enzyme activities and leaf litter decomposition in a wet tropical forest. *Soil Biology and Biochemistry* 64: 89-95.
101. Wei, Y, P. (2001) Seasonal Dynamic Study on the Amount and Nutrient of Litter of Pinus taeda Plantations in the High-Elevation Area. *Jour of Fujian Forestry Sci and Tech*. 28(3) 62-65 (in Chinese with English abstract).
102. Wei, Z, S. (2017) Responses of Decomposition of Flaveria bidentis litters to Simulated Nitrogen Deposition (in Chinese with English abstract).
103. Wei, Z, S., Li, H, Y., Li, K, L., Yang, D, L.,Huangfu, C, H. (2017). Effects of simulated N deposition and burial on Flaveria bidentis litter decomposition and nutrient release. *Chinese Journal of Ecology*. 36(9), 2412-2422. (in Chinese with English abstract).
104. Xing, Jun, Tian., Hiroshi, Takeda., Tatsuo, Ando. (1998). Dynamics of carbon and nutrients in Abies needle and Betula leaf litters during the two-year decomposition period in a subalpine coniferous forest. *The Society of Applied Forest Science*. 1998(7).
105. Xiong, W, D., Zhao, Y, T., Lu, C, Y., Lin, P. (2004). Seven metal element's biological accumulation and circulation of sonneratia apetala plantation in leizhou, Guangdong. *Chin J Appl Environ Biol*. 10(1), 027-034. (in Chinese with English abstract).
106. Xu G, Hu Y, Wang S, Zhang Z, Chang X, Duan J, Luo C, Chao Z, Su A, Lin Q, Li Y, Du M (2009) Effects of litter quality and climate change along an elevation gradient on litter mass loss in an alpine meadow ecosystem on the Tibetan plateau. *Plant Ecology* 209: 257-268.
107. Xu X (2006) Nutrient dynamics in decomposing needles ofPinus luchuensisafter typhoon disturbance in a subtropical environment. *Annals of Forest Science* 63: 707-713.
108. Xu X, Hirata E, Shibata H (2004) Effect of typhoon disturbance on fine litterfall and related nutrient input in a subtropical forest on Okinawa Island, Japan. *Basic and Applied Ecology* 5: 271-282.
109. Xu X-n, Shibata H, Enoki T (2006) Decomposition patterns of leaf litter of seven common canopy species in a subtropical forest: dynamics of mineral nutrients. *Journal of Forestry Research* 17: 1-6.
110. Xu, X, N., Eiji Hirata., Tssutomu Enoki., Youshihiro Takashiki. (2004). Leaf litter decomposition and nutrient dynamics in a subtropical forest after typhoon disturbance. *Plant Ecology*. 173(2) 161-170.
111. Yan, S, W., Chen, A, M., Lin, Y, M., Sun, F., Deng, H, J., Du, K., Wu, C, Z., Heng, B, T. (2017). Comparisons on content and reabsorption rate of nutrients in leaf of Eucalyptus camaldulensis at different stand ages in arid-hot valley and their linear-regression analysis. *Journal of Plant Resources and Environment*. 26(1) 39-46 (in Chinese with English abstract).
112. Yang N, Butenschoen O, Rana R, Kohler L, Hertel D, Leuschner C, Scheu S, Polle A, Pena R (2019) Leaf litter species identity influences biochemical composition of

ectomycorrhizal fungi. *Mycorrhiza* 29: 85-96.
113. Yang, Keum, Chui., jae, Kuk, Shim. (2003). The Decomposition of Leaf Litters of Some Tree Species in Temperate Deciduous Forest in Korea. *Korean journal of ecology*. 26(6), 313-319.
114. Yu X, Chao L, Zhang W, Chen L, Yang Q, Zhang G, Wang S (2019) Effects of inorganic nitrogen and litters of Masson Pine on soil organic carbon decomposition. *PLoS One* 14: e0222973. doi: 10.1371/journal.pone.0222973.
115. Yu, Y, W., W, J, S. (2004). Dynamics and Decomposition Characteristics of Litter of Evergreen Broad-Leaved Forest with Schima Superba. *Journal of Soil and Water Conservation*. 18(2), 63-65. (in Chinese with English abstract).
116. Yuan F, Wang Y-Y, Li M-J, Jiang C-Y, Liu H-N, Li K-L, Hong T, Wu C-Z, Chen C (2020) Dynamic characteristics of metal element content and return of Casuarina equisetifolia litter at different distances to the coastline. *Chinese Journal of Plant Ecology* 44: 819-827.
